# Poverty and family resilience in Belém-Pará

**DOI:** 10.1186/s41155-021-00176-x

**Published:** 2021-04-23

**Authors:** Larissa Araújo Matos, Edson Marcos Ramos Leal, Fernando Augusto Ramos Pontes, Simone Souza Costa e Silva

**Affiliations:** Universidade Federal do Pará (UFPA), Pará, Brazil

**Keywords:** Poverty, Family, Resilience

## Abstract

Family resilience is a complex, multi-determined behavior caused by the inseparable action of risk and protection factors. The purpose of this paper is to associate aspects of family resilience with multiple dimensions of poverty through a quantitative, descriptive, correlative, exploratory study with a sample of 448 low-income families in thirteen Social Assistance Reference Centers in Belém, Pará. The instruments used in the study were the Family Resilience Profile Questionnaire, the Social and Demographic Inventory, and the Family Poverty Rate. The results state that the families are not living in extreme poverty; however, they still face adversities due to the poverty. A significant presence of women, where 90.6% of the participants were mothers living in a single-parent family, attests that women are still the part of the population most affected by poverty. Furthermore, the results showed that the higher the poverty level, the lower the family resilience, and aspects such as work, knowledge and human development, especially child development, are aspects that enhance family resources to face adversities.

## Introduction

The interest for dealing with adversity whether by individuals or groups has been the object of interest of psychology. Empirical evidence has suggested that overcoming the obstacles seems to happen because of the action of multiple factors such as the intensity of the adversities in social, economic, cultural, and historical contexts, the strategies for handling and adapting to the difficulties faced, and the support received. Thus, these aspects have to be investigated and analyzed carefully, to understand how individuals and groups develop in the environment where they are (Pessoa, Coimbra, Koller, & Ungar, 2018; Wright, Masten, & Narayan, 2013).

People and groups’ capacity of handling failures and setbacks has been called resilience by psychology. This concept had several changes over time, but had a boost with the movement lead by Martin Seligman in the 1990s globally known as positive psychology. The purpose of this movement was to study the potential of the human being, i.e., “what works” for people (Seligman & Csikszentmihalyi, 2000).

This positive perspective that seeks to identify positive, healthy expressions of individuals and groups that are in disadvantage is evident in the initial studies on resilience carried out with children who, despite their vulnerability history, experienced a healthy development. However, the results of these researches fostered the notion of resilience as a phenomenon equivalent to the notion of invulnerability, a characteristic from the individual. Nonetheless, as the researches evolved, the term “invulnerable” has been replaced by more suitable terms such as stress resistance and resilience (Pessoa et al., 2018; Wright et al., 2013).

Overcoming the notion of invulnerability brought other assets for the resilience studies, helped understand that overcoming adversities is not an individual process, but a phenomenon that takes place in groups; therefore, there is a point in talking not only about individual resilience, but in family resilience as well. Besides having a dual dimension, individual and familiar, the researchers in the area recognized that this concept that involves two distinct criteria: (1) the presence of a significant threat to the development of the individual, and (2) despite the risk exposure, the adaptation of the individual or system is satisfactory by a set of criteria, being a complex, multi-determined process (Wright et al., 2013).

Knowing the mechanisms used by these families can be useful in interventions at systems that face crisis and that cannot develop efficient strategies. McCubbin and Patterson (1983) create the double ABCX model, to identify the multiple variables that explain the differences observed among families adapting to stressful situations.

McCubbin and Patterson (1983) described the double ABCX model, where the A factor corresponds to an event or transition that affects the family unit, which produces or has the potential to produce changes in the family system. A good example is the loss of a job, the unexpected death of a family member, a birth of a person with disabilities, in other words, events that the family does not expect, and that will require role and behavior changes from everyone, to adapt to the challenges faced (McCubbin & Patterson, 1983).

The B factor corresponds to the resources that the family develops in response to a stressor event, being the capacity the family has to prevent an event that generates changes in the family social system from creating a crisis or a disturbance in the system. The resources than become part of the family’s capacity to face the crisis, and may be classified in three ways: (a) individual resources such as personality traits, physical and emotional health, self-esteem, and intelligence; (b) family resources such as cohesion, adaptability, organization, communication, and problem-solving; and (c) community resources, concerning the capability of family to call the supportive network available by the community. Such resources reveal the capacity of the family to change their course of action in face of the obstacles found (McCubbin & Patterson, 1983).

The C factor in the ABCX model is the definition that the family gives to the stressor event that it meets. It is, therefore, a subjective definition attributed by the family at difficult moments and how these affect the group. This subjective meaning reflects the representations, the family values, and its previous experience when dealing with the changes and struggling the crisis. The perspective of a family can vary when perceiving the changes and relocations in life as challenges to face, or interpreting a stressor event as uncontrollable and a prelude for the family’s death (McCubbin & Patterson, 1983).

The crisis (the X factor) was concealed as a continuous variable that denotes the quantity of pile-up, disorganization, or maladaptation of the family social system. Differently from the stress, which is an imbalance, crisis is the incapacity to restore its stability and the continuous pressure to make changes in its structure and interaction patterns. In other words, the stress may not become a crisis if the family handles the resources available to resist the changes, thus maintaining family stability (McCubbin & Patterson, 1983).

McCubbin and Patterson (1983) proposal on the adaptation process is appropriate with the current literature, which addresses the concept of resilience as a complex, multi-determined process caused by the inseparable action of risk and protection factors (Oliveira & Morais, 2018). In fact, an attentive look at the variables of the double ABCX model reveals that the adaptation process involves the dynamic games between the forces that boost or hinder the overcoming of adversities. In this sense, poverty is a threat to the family development and functioning, causing stress to their members and, consequently, the need to struggle the adversities arising from its presence.

Poverty is a condition that exposes the people to social and emotional disadvantages and may cause several forms of conflict in family relationships (Fortini, Teixeira, Silveira, & Moreira, 2019; García, Gómez, Gómez, Marín, & Rodas, 2016), inadequate access to public services, poor educational and social and economic levels, increasing the vulnerability of children and adults. Living in poverty is beyond the lack of financial resources (Araújo & Flores, 2017), and it is essential to understand it as a multidimensional phenomenon (Barros, Carvalho, & Franco, 2006; Silva et al, 2017).

Although the literature recognizes that poverty is a multidimensional phenomenon, i.e., which involves a set of characteristics and dimensions in the social and private life, due to the difficulty to measure and encompass the several dimensions of poverty, the one-dimensional analysis of the phenomenon by focusing on the poor family income has prevailed. In this sense, income has been considered one of the main determinants of neediness in families, and, therefore, is a strong candidate to measure the poverty (Barros et al., 2006).

The instrument developed by Barros et al. (2006) is a multidimensional poverty index known from Pesquisa Nacional por Amostra de domícilio (PNAD) information. The index, in addition to the possibility of being classified at the level of each family, has characteristics that make it additively aggregable. It is composed of six dimensions, 26 components, and 48 indicators, where the dimensions are the following: Vulnerability, Access to knowledge, Access to work, Family income, Child development and Housing needs. There are 48 questions for families, who must answer yes or no. Each yes is counted as an unsatisfied need, a need, or a source of vulnerability and, therefore, leads to the poverty indicator increasing the family's score towards a greater degree of poverty.

From the instrument, it is possible to understand how these dimensions elaborated by the authors influence the concept of multidimensional poverty. For example, the vulnerability dimension assesses the amount of additional resources that a family needs to satisfy its basic needs, in relation to what would be required by a “standard family.” The presence of, for example, pregnant women, children, adolescents, young people, and the elderly increases the vulnerability of families as the volume of resources per capita needed to satisfy their basic needs grows (Barros et al., 2006).

Access to knowledge, according to the authors, is the most important dimension for a family to be able to access resources to satisfy their needs. In other words, education, professional qualification can help families out of poverty, as well as access to work, as they provide families with conditions so that they can exercise their knowledge in practice and thus have financial resources to meet their needs. Family income consists of the understanding that the vast majority of a family's basic needs can be satisfied through goods and services purchased on the market, family income per capita becomes a fundamental resource. It is important that families have resources so that they can satisfy their demands, this income can be accessed through autonomous means or also through income transfers from the government (Barros et al., 2006).

The last two dimensions are child development and housing. Child development is linked to the concept of multidimensional poverty, inasmuch as, every society has as goals to guarantee children conditions for their development, thus, when children are out of school or subjected to child labor the chances of remaining in the cycle of poverty they are greater, as well as the reverse. And finally, the housing conditions of a family have a close relationship with health issues, if the family has access to basic sanitation, clean water, garbage collection, adequate structure for shelter, among other elements are essential for families not to remain in poverty conditions (Barros et al., 2006).

In a one-dimensional perspective, there are no connections established between the people’s income and other dimensions of their lives as if they did not exist or were not relevant to understand poverty and the means to overcome it. Such multidimensional terms consider aspects such as the access to basic services, the capability of individuals to choose and follow the paths they appreciate, characteristics such as dignity and empowerment, the insertion of individuals into the society, among other aspects (Silva, Bruno, Silva, & B, N., 2020).

In this sense, there are privations ranging from the lack of access to health care services and public education to social and cultural provisions that difficult certain social groups to have the life they want to live. Income becomes a mean, among others, which makes possible the free development of the individuals and not as an end in itself. In modern societies, the look at the inequality cannot ignore the needs to overcome the asymmetry of access to goods and services. An expressive portion of the population has been living at bay of minimum living conditions, poverty is not only the state of a person needing material goods; it also corresponds to a specific inferior, unappreciated social status that leaves a deep mark on the identity of the people who faced such experience (Campello, Gentili, Rodrigues, & Hoewell, 2018).

Data on poverty in Brazil points out the North and Northeast regions are the most affected by high poverty and neediness rates. According to information from the Brazilian Institute of Geography and Statistics IBGE, 2019, extreme poverty in Brazil affects 13.5 million people having an income of up to 145 Reais per month, a record in the latest seven years. The growth of unemployment, leaner social programs, and the lack of adjustment in subsides such as the Bolsa Família increased the portion of the poorest people (IBGE, 2019).

In Pará, almost half the population (46%) was living below poverty line in 2017 according to the survey conducted by IBGE (2019). Such population in the state of Pará earned less than R$ 406 a month. The state with the third largest percentage on the North region stays behind only the states of Acre and Amazonas. Besides such data, in 2019, the then-president Ministry of Citizenship pointed that 20.97% of the population of Belém was living in extreme poverty, corresponding to 311,525 people. Such data is an evidence that, in a country with alarming levels of social inequality, basic rights denied, where at least 65% of the population was denied to at least one right (IBGE, 2019), the role of the State in assuring minimum conditions is essential so that families can meet their demands.

Evidence state that poverty conditions, especially chronic ones, affect human development and generate a “chain effect”, causing a series of stressful episodes in the life of the individual or family system, making it more difficult to properly respond to these events, which increases the powerlessness and the levels of stress, and makes this cycle continuous (Silva, Cunha, Ramos, Pontes, & Silva, 2019).

Despite the adversities, some factors contribute with the overcoming of risk situation by families such as those caused by poverty. Therefore, it should be considered that, in the background where the families develop, there are other elements, which implies recognizing and investigating the presence of factors that can protect the human being and mitigate the negative effects of the risks caused by poverty (de Lira & de Morais, 2020).

Therefore, this paper associated the Family Resilience Profile Questionnaire, according to Peixoto and Martins (2012) and the six dimensions of the Family Poverty Rate presented by Barros et al. (2006), with families living in poverty conditions in the city of Belém (PA).

## Method

### Participants

Four hundred forty-eight mothers/people in charge of low-income families in the city of Belém (Pará) participated in the study. The criteria for the inclusion of families were: (a) Families registered with the Cadastro Único of the Federal Government until August/2015. The Cadastro Único is a register that allows the government to know who low-income families are and how they live in Brazil. It was created by the Federal Government, but is operationalized and updated by city halls for free. Through Cadastro único, it was possible to identify poor families in Belém-PA.

### Environment

The research studied the 12 Social Assistance Reference Centers (CRAS), located in suburban areas in Belém in the neighborhoods of Aurá, Barreiro, Bengui, Cremação, Icoaraci, Guamá, Jurunas, Mosqueiro, Outeiro, Pedreira, Terra Firme, and Tapanã.

### Instruments

#### Social and demographic inventory (ISD)

The Social and Demographic Inventory intends to characterize the families participating in the study. It is traditionally used by the research group at the Development Ecology Laboratory, comprising 41 items (Silva, Pontes, Lima, & Maluschke, 2010), being the first contact with the families and making possible the relationship between researcher and researche. The present study surveyed the information concerning the family status, marital status, labor status, age, education, and quantity of children of the participants, besides information on their family structure and participation at the Programa Bolsa Família (PBF).

#### Family poverty rate (FPR)

The instrument developed by Barros et al. (2006) is a multidimensional poverty index known from PNAD information. The index, in addition to the possibility of being classified at the level of each family, has characteristics that make it additively aggregable. The article concerned did not present Cronbach’s alpha. However, this instrument has been used in Brazil since 2006 in different studies in psychology and other areas (Fraga, Filho, Coronel, & Vieira, 2017; Gomes, Ribeiro, & Mendes, 2014; Marin & Ottonelli, 2008; Silva et al., 2019). And is an important instrument used by the Federal Government to obtain information on how multidimensional poverty affects Brazilian families in this situation.

It is composed of six dimensions, 26 components, and 48 indicators, where the dimensions are the following: Vulnerability, Access to knowledge, Access to work, Family Income, Child development, and Housing needs*.* There are 48 questions for families, who must answer yes or no. Each yes is counted as an unsatisfied need, a need, or a source of vulnerability and, therefore, leads to the poverty indicator increasing the family's score towards a greater degree of poverty.

In this study, each dimension was evaluated separately, and its variation can range from 0 (zero—for families without any trait of poverty) to 100 (one hundred—for absolute poor families). The descriptive statistics and poverty levels used for the present sample are in Table 1. Since the instrument does not have standardized cutoff points, the results were divided into the following quartiles: poorer families (25% poorer), families in average poverty (families ranging from 26% to 75%) and less poor families (25% less poor).

**Table 1:** Statistics and categories concerning the Family Poverty Rate and their dimensions in poor families in the city of Belém (PA)

Dimensions	Statistics			Levels		
	Mean (SD)	Min.	Max.	Low poverty	Medium poverty	High poverty
Vulnerability	4.16 (1.31)	1	10	1 to 3	3.01 to 4.99	5 to 10
Knowledge	2.43 (1.19)	0	6	0 to 2	2.01 to 2.99	3 to 6
Work	2.85 (1.33)	0	6	0 to 2	2.01 to 3.99	4 to 6
Income	0.51 (0.87)	0	3	0 to 0	0.01 to 0.99	1 to 3
Development	0.54 (0.75)	0	4	0 to 0	0.01 to 0.99	1 to 4
Housing	3.91 (1.71)	0	9	0 to 3	3.01 to 4.99	5 to 9
IPF total	14.40 (3.89)	5	27	5 to 12	12.01 to 16.99	17 to 27

Source: Development Ecology Laboratory Database

#### Family resilience profile questionnaire (QPRF)

This instrument intends to identify the family resilience profile. It was translated by Peixoto and Martins (2012) and applied with families in Portugal. It comprises 50 items distributed in the following five dimensions: family changes, family coherence, family flexibility, family involvement, and family social support. Therefore, the instrument intends to obtain information on family resilience from such five scales, individually and qualitatively. The original instrument built by McCubbin and McCubbin (1993) is used in different studies in other countries and made it possible to produce relevant data on resilience in different risk situations (Alianiello, 2005; Yeh & Bull, 2012; Hall et al., 2012;

In the course of the research, it was observed that there were no instruments that measure resilience in Brazil, therefore, the choice of the instrument validated by Peixoto and Martins (2012), at that time was the possible choice in view of the limitation and despite the instrument not being validated for Brazil, he still contributes relevant data to the theme of the manuscript. And before conducting the collection, A pre-test was carried out with some subjects, from the researchers’ social background, with a similar profile of the study participants. Care was taken to apply this instrument to people with a similar profile to see if the language was understandable for them. The change made was in relation to data analysis because it was noted the need to perform some adjustments in the instrument such as standardization of scales. Since it does not have standardized cutoff points, the results were divided into quartiles under the following classifications: families with low resilience (corresponding to the quartile 1 or the 25% less resilient) families with medium resilience (corresponding to the quartiles 2 and 3 or families located between 26% and 75%), and more resilient families (corresponding to the quartile 4 or the 25% more resilient families).

### Collection procedure

The development of this research took place concomitantly with other researches within the macro project “Poverty and Development Ecology”. Initially, the macro project was submitted to the Ethics Committee on Scientific Research at the Center of Tropical Medicine of the Federal University of Pará, which approved it under the opinion CAAE 21653814.4.0000.5172.

It was agreed that the participants’ criteria would be the registration at the *Cadastro Único* (CadÚnico), whereas the families registered under this system are poor or in economic and social vulnerability. The research then followed the following stages: 1st stage: the request, to the National Office of Income and Citizenship (SENARC/ MDS) of the Ministry of Social Development and Fight Against Hunger (MDS), of the database concerning families registered at the CadÚnico in the city of Belém, until August/2015, and further sample calculation of the quantity of participating families per neighborhood in Belém, based on the population of 154,779 registered at the CadÚnico, through simple and stratified sampling procedures; 2nd stage: the selection of the instruments applied; 3rd stage: pilot study with families whose profiles are similar to those of the participants. After the pilot study, it was decided to make collections through individual interviews, to facilitate the suppression of ideas; 4th stage: contact with the Fundação Papa João XXIII (FUNPAPA), requesting the authorization for interviews at the CRAS. With the authorization granted, the coordinators at the CRAS were contacted and presented to the research and its procedures, and requested to inform the days of attendance to people with demands of CadÚnico/Programa Bolsa Família, not participating in other activities offered by the center.

At the CRAS, the 6th stage consisted of the division of the team in groups of two for collecting the data, the groups addressing to an individual who could be a possible participant, introducing themselves and asking: (a) What was the reason that made her go the CRAS, (b) Which neighborhood did her live, and (c) If she had children or children under her responsibility at the age between 5 and 18. Once the responses to the questions were according to the requirements of the research, the participant’s consent was requested for the interview by reading and signing the Term of Free and Clarified Consent. The average duration of the interview was one hour, and the data collection of the 448 families took part throughout the year 2016.

### Analysis procedures

To conduct the analysis, the data was obtained from a file at the SPSS 20.0 software. The results of the Family Resilience Profile Questionnaire and the Family Poverty Rate considered the sample quartiles method (Agresti & Finlay, 2012). In order to associate the dimensions of the QPRF and the FPR, Correspondence Analysis was performed, which is an exploratory statistical method for verifying associations or similarities between qualitative variables or categorized continuous variables (Fávero, Belfiore, Silva, & Chan, 2009). The associations between the categories are deemed moderately significant when the confidence coincidence value is 50 ≤ *γ* × 100 < 70%, and strongly significant, when the confidence coefficient value is (*γ*) ≥ 70.00%. Correspondence analysis was performed with the aid of the Statistica application version 6.0. In all the tests, α was equal to 5% (*p* ≤ 0.05) for the rejection of null hypothesis.

## Results and discussion

This section is presented in three topics. The first topic corresponds to the general social and demographic data of the participants, the second one brings the general results of families at the Family Poverty Rate (FPR), and, finally, the association between the data obtained with the Family Resilience Profile Questionnaire (QPRF) and the Family Poverty Rate (FPR).

## Social and demographic characteristics of participants

This section will present the results of the sociodemographic data. Table 2 shows important information.

**Table 2:** Sociodemographic characteristics of poor families living in the municipality of Belém (PA) (*n* = 448)

**Family status**							
Mother	Father		Grandmother	Other		Uninformed	
406 (90.6%)	14 (3.1%)		13 (2.9%)	12 (2.7%)		3 (0.7%)	
**Marital Status**							
Married/stable union	Single		Divorced/ Separated	Widows		Uninformed	
202 (45.1%)	206 (46%)		30 (6.7%)	7 (1.6%)		3 (0.7%)	
**Family Type**							
				Single parent			
Nuclear intact	Headed by grandparents	Reconstituted	Extended	Female	Male	Headed by grandparents	Extended
132 (29.4%)	12 (2.6%)	25 (5.5%)	28 (6.2%)	145 (32.3%)	4 (0.8%)	16 (3.5%)	86 (19.1%)
**Employment Situation**							
Employed				Unemployed			
259 (57.8%)				189 (42.2%)			
**Beneficiary of the PBF**							
Yes				No			
411				37			
**Age**							
20-29	30-39	40-49	50-59	60-69	70-79	Uninformed	
88 (19.6%)	217 (48.4%)	98 (21.8%)	32 (7.1%)	8 (1.7%)	1 (0.2%)	4 (0.8%)	
**Schooling**							
EFI	EFC	EMC		ESC	Illiterate	Uninformed	
97 (21.6%)	109 (24.3%)	229 (51.1%)		7 (1.5%)	4 (0.8%)	2 (0.4%)	
**Number of children**							
1 Child	2 Children	3 Children		4 Children	5 Children	6 Children	
105 (23.4%)	183 (40.8%)	103 (22.9%)		43 (9.5%)	8 (1.7%)	6 (1.3%)	

The results pointed that the majority of the participants was mothers 90.6% (*N* = 406), the most part 46% (*N* = 206) was single, the main family type was female single parent 32.3% (*N* = 145), the main age range of the tutors was 30-39 years old with 48.4% (*N* = 217), and the majority 40.8% (*N* = 183) had two children. About the working situation of the participants, the data pointed that 54% (*N* = 244) had a job, 50% of them being domestic workers. Because of the excessive load and responsibility in different roles, the family income tends to be low, ranging from 1 to 2 minimum wages 45.19% (*N* = 202).

The results stated that the female presence was solid in the present study and the family type, predominantly female single parent. Data from the IBGE (2019) showed that the households whose head is a woman went over 30 million in 2017, being 30.5 million or 28.5% of the total of Brazilian households. In 2012, the percentage was 22.7% or 23.26 million households. Therefore, the female single-parent family is a reality present in the society, and, as in the other families, those face several adversities as well. Women seem to be more affected by adverse situations such as double time working, the difficulty to have jobs with better remuneration, and the lack of support of the former partner to share responsibilities, these factors can make these women/mothers feel overloaded due to the excess of functions (Cúnico & Arpini, 2014).

About the quantity of children, the study pointed that the majority had children, this data reflects a tendency in the current Brazilian scenario, and it is essential to observe the significant changes faced by the family systems, associated with transformations of demographic, social, and cultural nature. Among the transformations of demographic order, the decrease in fertility and the aging of the population stand out. Data from the IBGE in 2017 points that Brazil has faced a reduction in the number of children per woman, which has happened progressively in the latest years.

Other data reveals that the majority of the participants has a job; however, in general, women from lower classes are employed in the informal market and had poor working and wage conditions. Furthermore, when there is no social and emotional support from the fathers of those women’s children, they begin to exercise the main role of looking after these individuals and, when they need to quit their jobs, either formal or not, they do it even at the risk of impairing their quality of life and that of their children (Costa & Marra, 2013).

Other result that draws our attention is that 43.85% (*N* = 196) were living with less than one minimum wage per month, i.e., the economic and social conditions of a significant portion of the sample was below the minimum required to having access to basic goods and services, thus justifying the results that 91.74% (*N* = 411) of the sample was beneficiary of the Programa Bolsa Família. In view of that, data from the IBGE (2019) reveal that 50% of Brazilian workers receive on average 15% less than the minimum wage. Furthermore, the income of those who earn more is 360 times above that of the workers who have lower incomes, i.e., these data state how Brazil is still a country with high social inequality levels.

About the education, the majority of the participants had completed secondary education 51.1% (*N* = 229). This is a positive data, since that, according to the Continuous National Household Sample Survey (Pnad) of 2018, more than half the Brazilians at the age of 25 or more did not complete basic education. Comparing the participants of this study with the data from the IBGE, the participants were at a satisfactory educational level.

### General family results in the family poverty rate (FPR)

Despite all the participants of this research were considered poor, since they were registered at the CadÚnico, it was observed that, according to the IPF, about 80% of the participants had a family poverty level ranging from 20% to 40%. Graph below demonstrates the result of the family general IPF.

**Fig. 1:**
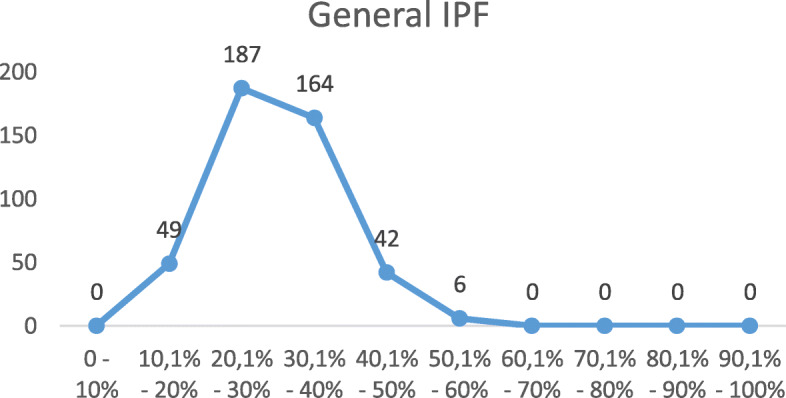
General IPF

Figure 1 demonstrates that about 80% of the participants, which corresponds to 351 families, presented a family poverty level ranging from 20% to 40%, which puts them into a not extreme social and economic vulnerability level, below the average established by the instrument. In parametric terms, the national IPF average was 25 % in 2003 (Barros et al., 2006).

This data provides the reflection that there is the possibility that social protection systems, transfer payment to individuals or families, also associated with other compensatory measures, especially amidst the education, health, and labor policies, have an important role of breaking a cycle that ravages an expressive portion of the Brazilian population in the reproduction of poverty (Dantas, Miranda, Dusek, & Avelar, 2018). Data from 2017 of the former Ministry of Social Development pointed that 39% of the population in Pará had as their main source of income the amounts transferred via the social program.

Although, it is worth emphasizing that poverty is a multidimensional phenomenon, and its analysis should not be made in function of the means and resources owned by the individuals. However, in Brazil, the main Brazilian transfer payment program does not only relieve the financial difficulties of the beneficiary families, but also provides the access to basic rights such as health care, education, and work.

The Programa Bolsa Família (PBF) brings positive effects in the human development of the beneficiaries, especially among the younger generations, even it is not sufficient to ensure human development in its dimensions. Public policies for placement in the labor market for employment and income and the policies for social inclusion and assurance of rights need to the transversal and integrated to the PBF, and it is undeniable the modifications that the program has brought to its beneficiaries (Dantas et al., 2018)

### Correspondence analysis between the family resilience profile questionnaire (QPRF) and the family poverty rate (FPR).

This topic presents the results of the association between the Family Resilience Profile Questionnaire and the Family Poverty Rate of the participants in the study. The data is presented in Table 3 below.

**Table 3:** Associations between Family Resilience and Family Poverty Rate

Variable		IPF total		
	Category	Low poverty	Medium poverty	High poverty
Total family resilience	Low	0.55(41.91)	− 0.45(0.00)	− 0.06(0.00)
	Moderate	0.02(1.33)	− 1.44(0.00)	1.72(91.44)*
	High	− 0.17(0.00)	2.10(96.47)*	− 2.35(0.00)

Note: Statistics from the Correspondence Analysis Note 2:*significant association between total family resilience and medium poverty and high poverty

Table 3 demonstrates that IPF and QRPF are related in such a way that the higher the poverty, the lower the family resilience. On the other hand, the data state that the lower the poverty, the higher the family is likely to present better resilience levels, which can be explained by less requirements or stressing events related to social and economic aspects so that the families have better conditions to handle their daily adversities (de Lira & de Morais, 2020).

The data suggest the families can add resources to the family system, protecting them in face of difficult situations. It is possible that family groups who present good resilience levels fall under the profile identified by McCubbin and McCubbin (1993) as rhythmic and regenerative family. Rhythmic families are those who, despite the adversities, promote the development of predictable activities and routines within the family unit encompassing the entire family group in an effort to share a purpose and meaning of union, regularity, and predictability. Regenerative families are those that, when feeling in the control of their lives, can influence either what is good and what is bad, by taking an active position in face of the difficulties, encouraging their members to seek new approaches for the problems, and, consequently, become able to take relevant decisions (McCubbin & McCubbin, 1993)

Moreover, one might think that resilience develops gradually from a sequence of proximal process with crescent complexity faced since the beginning of life, which makes possible that, even in high-risk backgrounds, the people, families, or communities handle the adversities they face to find answers to their problems and needs. It is, therefore, a complex phenomenon built in the intersection between the multiple backgrounds with which the human being interacts directly or indirectly and that whose presence is observed more clearly when facing an adverse situation, either temporary or constant (Gaspar & Balancho, 2017).

### Correspondence analysis between the family resilience profile questionnaire (QPRF) and the six dimensions of the family poverty rate (FPR).

This topic brings the results of family resilience analysis and the dimensions of family poverty rate in Belém, enabling associations on the existing relationships between these variables. The results obtained are in Table 4.

**Table 4:** Associations between Family Resilience and the dimensions of Family Poverty Rate (FPR)

Dimension of FPR		Family Resilience		
		High	Average	Low
Vulnerability	Low	− 0.97(0.00)	− 2.36(0.00)	0.96 (0.00)
	Average	− 0.27(0.00)	0.13(10.52)	0.15(12.10)
	High	0.64(47.79)	0.45(35.08)	2.78(99.45)*
Lack of access to knowledge	Low	1.06(71.29)*	0.45(35.08)	− 1.24(0.00)
	Average	− 0.96(0.00)	0.45(35.08)	0.45(35.08)
	High	0.96(66.26)	1.24(78.56)*	− 1.37(0.00)
Lack of access to work	Low	2.16(96.96)*	0.17(13.12)	− 2.66(0.00)
	Average	− 1.82(0.00)	1.29(80.27)*	0.64(47.71)
	High	1.91(94.45)*	− 1.82(0.00)	− 0.16(0.00)
Lack of access to income	Low	*p* = 0.081		
	Average			
	High			
Child development deficit	Low	− 0.42(0.00)	1.46(85.51)*	0.51(38.80)
	Average	− 1.24(0.00)	− 1.75(0.00)	0.45(35.08)
	High	2.27(97.66)*	− 1.89(0.00)	− 1.37(0.00)
Housing deficit	Low	*p* = 0.431		
	Average			
	High			

Note: Correspondence Analysis Data between instruments. Note 2: the symbol (*) represents all the significant associations found in the study among the studied variables

Table 4 states that some dimensions (Income and Housing) of the Poverty Rate (IPF) did not have significant associations with Family Resilience. The dimensions *Vulnerability*, *Knowledge*, *Work*, and *Development* had associations with the Total Family Resilience

The results pointed the existing relationship between Vulnerability and Family resilience, where families that are more vulnerable are likely to have low resilience scores. The dimension *Vulnerability* represents the resources that a family needs to meet its basic needs about what a “standard family “would require (Barros et al., 2006). Some elements such as the presence of pregnant women, children, adolescents, youths, and elderly can increase the vulnerability of families as the volume of resources per capita required for meeting their basic demands grow. For instance, this dimension states that the presence of the mother is particularly important. If others are raising the children, these infants have a higher probability of falling unprotected, working in painful activities, or being out of school or ill without adequate medical attention.

Consequently, it is possible to say that the families participating in the study had several needs, as they present high vulnerability levels that justify the poor resilience scores. These families are not likely to have resources, either individually, in family, or in the community, which may help them meet their needs, i.e., to overcome their daily adversities (McCubbin & Patterson, 1983). The scarcity of resources for a long time disorganizes or incapacitates the family system from developing effective strategies for handling the adversities.

Despite the vulnerability level of the families investigated, the data obtained with the QPRF stated that the majority of them fell under the moderate resilience level (Table 4), leading the conclusion that these families, despite the several daily demands, could construct resources over time and manage them in face of the adversities imposed by the poverty (McCubbin & Patterson, 1983). According to the double ABCX model, the families reconstruct their resources when facing the challenges, the family system members are capable of reinventing themselves by finding new action strategies, new ways to face the challenges that arise in crisis situations such as those imposed by the poverty (McCubbin & Patterson, 1983).

The analysis revealed that one of the resources driven by the families is *Knowledge.* Families with higher knowledge scores presented high resilience levels as well. Knowledge is a good example of a resource constructed by the family system over time as a response to the needs generated by the adversities (McCubbin & Patterson, 1983). Among all the means available that a family can have to meet their needs, access to knowledge is certainly one of the most important ones (Barros et al., 2006). Although the IPF restrains the knowledge to the notion of formal education, this dimension can be understood in a broader way with a view to the access to consistent and valid information that favors the confrontation of adversities.

The dimension *Work* had more association with resilience, where families with higher access to work had strong relationships with high and moderate family resilience. It is observed that this dimension stands out from the others when considering the set of aspects that comprises the concept of multidimensional poverty, namely: the lack of access to citizenship and basic rights such as food, health, education, employment, and income, the denial of the individuals to develop their capabilities, and the freedom so that the people can have a life that they appreciate (Sen, 2010; Silva et al., 2020). It is possible that such highlight justifies since work translates into resources that can be analyzed in two levels, both family and individual 1983).

The discussion around work as a family and individual resources implies retaking the discussion of the concept of poverty. Although we are dealing with the notion of poverty in a multidimensional perspective, we cannot disregard the role that income plays within the families (Barros et al., 2006). The income provides access to goods and services such as health and education to their members. The data found allowed us to consider that there was an increase of family income to the extent that the families had access to work, which in turn guaranteed family resources that had an impact on high and moderate family resilience levels.

Besides being considered a dimension that generates family resources that encourage the handling of the adversities arising from poverty, work must be understood as a dimension that has an impact on an individual basis. According to McCubbin and McCubbin (1993), individuals have resources, i.e., characteristics that work as instruments that will be used for struggling the challenges in crisis moments. In this sense, the positive association between work and resilience is feasible since being working develops feelings of competence and self-efficiency. By working, people have the opportunity to use their production capacity, thus generating some income for their families (Barros et al., 2006).

About *Child development*, the data showed that families with fewer difficulties in these dimensions were more likely to have high family resilience levels. Dimension Child development falls under four elements as per the Pnad, namely child labor, school dropout, scholastic backwardness, and child mortality. Such as in the other dimensions of the IPF, Child development is an object of assessment by the adult in charge of the family, its factors reveal the perception that adults attribute to the development of the younger generations. Considering this aspect allows grounds for many considerations already addressed in the literature, e.g., Matos, Santos, and Silva (2018) stated that their participants believed that, by studying, their children and grandchildren would have a better life in the future. They also observed that, despite the poverty they faced at that moment, the families considered education as a mean to provide opportunities to the younger generations.

The perception about the reality, i.e., the adversity, constitutes what McCubbin and Patterson (1983) called C factor, i.e., how people face the crisis, the adversities. The people’s perception has an impact on their behaviors on how they will handle such adversity. It is not absurd to assume that in families such as those participating in the present study, although they face difficulties due to poverty such as violence, unemployment, hunger, etc., they still believe in the development of the younger generations, which reveals higher family resilience levels (de Lira & de Morais, 2020; Torres et al., 2020).

The attentive look on how the dimensions investigated by the IPF and the data on family resilience, in face of the double ABCX model, allowed us to observe aspects that characterize the environment where and how people are living, encouraging family resilience. In general, the participants of the study, though they live under the continuous pressure of the adversities they face, can apply resources that make them handle their crisis, which attests the capabilities of these families to prevent the constant stressor events from causing devastating changes in the family system.

## Conclusion

The present study contributed with the literature by investigating family resilience on a population constituted of poor people, and identified, considering the poverty from a multidimensional perspective, the aspects that favor family resilience. It was observed that work, knowledge, and child development enhance the family resources, thus contributing with the overcoming of the adversities caused by poverty. On the other hand, vulnerability has an impact in the opposite direction since vulnerable families had poor resilience levels.

The study had limitations, one of the limitations of the study was in relation to the FPI and QPRF instruments. First, the authors who prepared it did not present the instrument’s Cronbach’s alpha, so it was not possible to be sure about the reliability of each dimension of the instrument, and the second was not validated for Brazil. It is considered that it will not be possible to relate the data obtained in the investigated metropolitan region with what is expected for Brazilian families in general. Despite this limitation, the data found in the present study can be analyzed in the light of national studies that used the same instruments or other similar measures.

However, psychology has a history of using instruments without validation in the country due to the difficulty of finding instruments that have already been validated, however, studies that use such instruments without validation are able to produce data and information of relevance to the area, as well as the instrument of measurement of the level of poverty used in the present research, which despite being limited by the absence of the Cronbach’s alpha, has been used since 2006 by different scholars of the theme and also by the Brazilian Government to promote social policies and understand how poverty presents itself in Brazilian families.

Another suggestion that the study points out is the need for studies to validate instruments for the country in order to remedy a lack of such instruments with this configuration in the studies of psychology.

Contributed to developmental psychology to understand how poverty can affect the phenomenon of resilience in families that are in this situation, although the instruments not being validated, they bring relevant information for understanding the profile of poor families in Brazil and which elements can favor the resilience of these groups. In addition, it contributes to public policies when it presents an instrument elaborated according to information from national data, PNAD, which makes it possible to understand poverty in the multidimensional context, in addition to income. Promoting information that can be useful in the design and implementation of social public policies.

Additionally, we suggest carrying out studies with poor populations that are not registered at the Brazilian government’s social programs since there are approximately 20 million Brazilian living in poverty that are not in the official records such as street population, people without documentation, immigrants, etc. It is possible that the study of these populations will add information that, in addition to those presented here, will allow better understanding of resilience in low-income families.

## Data Availability

The datasets used and/or analyzed during the current study are available from the corresponding author on reasonable request.

## Abbreviations

PNAD: Pesquina Nacional por Amostra de Domícilios
CadÚnico: *Cadastro Único*
CRAS: Social Assistance Reference Centers
FPR: Family Poverty Rate
ISD: Social and Demographic Inventory
QPRF: Family Resilience Profile Questionnaire
PBF: Programa Bolsa Família
Funpapa: Fundação Papa João XXIII
