# Correction to: Poverty and family resilience in Belém-Pará

**DOI:** 10.1186/s41155-021-00197-6

**Published:** 2021-10-25

**Authors:** Larissa Araújo Matos, Edson Marcos Ramos Leal, Fernando Augusto Ramos Pontes, Simone Souza Costa e Silva

**Affiliations:** Universidade Federal do Pará (UFPA), Pará, Brazil

**Correction to: Psicol. Refl. Crít. 34, 12 (2021)**

**https://doi.org/10.1186/s41155-021-00176-x**

Following publication of the original article (Matos et al., 2021), the affiliation detail for the authors was incorrectly given as ‘Endereço Institucional, Rua Augusto Corrêa, S/N., Belém-Pará 66,075–110, Brazil’ but should have been ‘Universidade Federal do Pará (UFPA), Pará, Brazil’.

The author affiliation has been updated in this correction article and the original article (Matos et al., 2021) has been corrected.

## References

[CR1] Matos LA (2021). Poverty and family resilience in Belém-Pará. Psicologia: Reflexão e Crítica.

